# Quantitative Trait Loci and Maternal Effects Affecting the Strong Grain Dormancy of Wild Barley (*Hordeum vulgare* ssp. *spontaneum*)

**DOI:** 10.3389/fpls.2017.01840

**Published:** 2017-10-30

**Authors:** Shingo Nakamura, Mohammad Pourkheirandish, Hiromi Morishige, Mohammad Sameri, Kazuhiro Sato, Takao Komatsuda

**Affiliations:** ^1^Institute of Crop Science, National Agriculture and Food Research Organization, Tsukuba, Japan; ^2^Plant Breeding Institute, The University of Sydney, Sydney, NSW, Australia; ^3^Institute of Plant Science and Resources, Okayama University, Kurashiki, Japan

**Keywords:** dormancy, germination, QTL, wild barley, maturing temperature, maternal inheritance, domestication, pre-harvest sprouting

## Abstract

Wild barley (*Hordeum vulgare* ssp. *spontaneum*) has strong grain dormancy, a trait that may enhance its survival in non-cultivated environments; by contrast, cultivated barley (*Hordeum vulgare* ssp. *vulgare*) has weaker dormancy, allowing uniform germination in cultivation. Malting barley cultivars have been bred for especially weak dormancy to optimize their use in malt production. Here, we analyzed the genetic mechanism of this difference in seed dormancy, using recombinant inbred lines (RILs) derived from a cross between the wild barley accession ‘H602’ and the malting barley cultivar ‘Kanto Nakate Gold (KNG)’. Grains of H602 and KNG harvested at physiological maturity and dried at 30°C for 7 days had germination of approximately 0 and 100%, respectively. Analysis of quantitative trait loci (QTL) affecting grain dormancy identified the well-known major dormancy QTL *SD1* and *SD2* (located near the centromeric region and at the distal end of the long arm of chromosome 5H, respectively), and QTL at the end of the long arm of chromosome 4H and in the middle of the long arm of chromosome 5H. We designated these four QTL *Qsd1-OK*, *Qsd2-OK*, *Qsdw-4H*, and *Qsdw-5H*, and they explained approximately 6, 38, 3, and 13% of the total phenotypic variation, respectively. RILs carrying H602 alleles showed increased dormancy levels for all QTL. The QTL acted additively and did not show epistasis or QTL–environment interactions. Comparison of QTL locations indicated that all QTL except *Qsdw-5H* are likely the same as the QTL previously detected in the doubled haploid population from a cross between the malting cultivar ‘Haruna Nijo’ and ‘H602.’ We further examined *Qsd2-OK* and *Qsdw-5H* by analyzing the segregation of phenotypes and genotypes of F_2_ progenies derived from crosses between RILs carrying specific segments of chromosome 5H from H602 in the KNG background. This analysis confirmed that the two genomic regions corresponding to these QTL are involved in the regulation of grain dormancy. Germination tests of F_1_ grains derived from reciprocal crosses between H602 and KNG revealed that the H602 strong dormancy phenotype shows maternal inheritance with incomplete dominance. These results provide new insight into the mechanisms regulating grain dormancy in barley.

## Introduction

The timing of germination plays a key role in plant survival. Many plants have evolved the ability to suppress germination, even under favorable conditions, a phenomenon termed seed (grain) dormancy (Finkelstein et al., 2008; Nonogaki, 2014; Rodríguez et al., 2015). Having some seeds remain dormant in case conditions turn unfavorable may have adaptive advantages for wild plants. By contrast, many cultivated plants have been selected for weak dormancy. For example, barley (*Hordeum vulgare* ssp. *vulgare*) is a major cereal crop in the Triticeae and has a wide range of grain dormancy levels (Takeda and Hori, 2007). In general, the wild progenitor of cultivated barley (*H. vulgare* ssp. *spontaneum*) has very strong grain dormancy, enabling it to survive various adverse environmental conditions. By contrast, malting barley cultivars have very low levels of grain dormancy, as they have been bred for simultaneous, rapid germination upon imbibition for malt production.

Many genetic and environmental factors affect seed dormancy and examining the genetic mechanisms governing the large difference in grain dormancy between wild and malting barley can identify natural mutations for dormancy that will enhance our understanding of the mechanisms by which dormancy decreased during and after domestication. This approach will also improve the development of barley cultivars with higher levels of tolerance to pre-harvest sprouting (PHS), which can cause devastating damage to yield and grain quality.

A number of grain dormancy quantitative trait loci (QTL) analyses in barley have been carried out, mainly using populations from various combinations of barley cultivars. These analyses have detected dormancy QTL on all seven chromosomes, and revealed two major dormancy QTL, *SD1* and *SD2*, located on chromosome 5H near the centromeric region and at the distal end of the long arm, respectively (Ullrich et al., 1992; Oberthur et al., 1995; Han et al., 1996; Thomas et al., 1996; Li et al., 2003; Edney and Mather, 2004; Prada et al., 2004; Zhang et al., 2005; Vanhala and Stam, 2006; Hori et al., 2007; Bonnardeaux et al., 2008; Ullrich et al., 2009; Hickey et al., 2012; Gong et al., 2014). To examine the genetic mechanisms underlying the striking difference in grain dormancy between wild and malting barley, a QTL analysis of grain dormancy was previously carried out using doubled haploid lines derived from a cross between the Japanese malting barley cultivar Haruna Nijo (HN) and the wild barley accession H602 (Hori et al., 2007). This analysis detected dormancy QTL on chromosomes 1H, 4H, and at the *SD1* (*Qsd1*) and *SD2* (*Qsd2*) loci on chromosome 5H; these QTL explained approximately 5, 5, 70, and 6% of the phenotypic variation, respectively (Hori et al., 2007).

Previous work indicated that *SD1* is a major regulator of dormancy in wild barley, and a recent study used map-based cloning to identify the causal gene of *Qsd1*, which encodes an alanine aminotransferase (AlaAT; Sato et al., 2016). The main cause for *Qsd1* was found to be a substitution at amino acid 214 of AlaAT, from a leucine (L) in the dormant allele to a phenylalanine (F) in the non-dormant allele. This substitution is caused by a single-nucleotide polymorphism (SNP) in exon 9 and is highly correlated with the dormancy phenotypes. Therefore, the naturally occurring L214F substitution seems to have been an important mutation that occurred in wild barley to produce the transition from the strong dormancy in wild barley to the weak dormancy in cultivated barley.

By contrast, another dormancy QTL may have been selected in cultivated barley to prevent PHS. *Qsd2-AK*, a major dormancy QTL located at the *SD2* locus was found using recombinant inbred lines (RILs) derived from a cross between the dormant Japanese cultivar Azumamugi (Az) and the Japanese malting barley Kanto Nakate Gold (KNG) (Nakamura et al., 2016). The causal gene of *Qsd2-AK* was identified as *MITOGEN-ACTIVATED PROTEIN KINASE KINASE 3* (*MKK3*) by map-based cloning (Nakamura et al., 2016). The causal sequence polymorphism, thought to be a naturally occurring mutation in the Az dormant allele, causes a non-synonymous substitution from Asparagine (N) to Threonine (T) at the 260th amino acid, and reduces MKK3 kinase activity. After cultivated barley reached East Asia from the Fertile Crescent several 1000 years ago, the N260T mutation may have improved barley adaptation to the climate in East Asia by preventing PHS, because in this region, the harvest season tends to overlap with the rainy season in the Asian monsoon. Therefore, this mutation seems not to be related to the transition from wild to cultivated barley. In fact, the wild barley H602 does not have N260T mutation, as it has the evolutionarily conserved N260. Therefore, we do not know whether *SD2* is also involved in the regulation of the strong dormancy in wild barley.

To understand the complex, multigenic mechanism that regulates dormancy, we need to study minor and major QTL. The resulting knowledge will provide useful information for fine-tuning the level of dormancy in cultivars to balance germination at a level that prevents PHS and allows simultaneous germination at sowing and/or malting. Moreover, the identification of other causal genes for dormancy QTL in barley could also aid in the reduction of PHS in related grain crops. For example, *MKK3*, the causal gene for *Qsd2-AK* in barley, is also the causal gene for the major dormancy QTL *Phs1* in wheat (*Triticum aestivum*; Torada et al., 2016).

In this study, we examined the dormancy of barley using a QTL analysis of RILs derived from a cross between wild barley H602 and cultivated barley KNG. We found that four QTLs including *SD2* and a novel QTL on chromosome 5H acted as the major factors determining the strong dormancy of H602. In addition, the strong dormancy seems to be maternally inherited. These results identify novel targets for future studies of the mechanisms that regulate dormancy in barley and wheat.

## Materials and Methods

### Plant Materials

A total of 94 F_9_ RILs were derived from a cross between H602 and KNG using a single-seed descent approach. H602 is wild barley (*Hordeum vulgare* ssp. *spontaneum*) accession. Kanto Nakate Gold (KNG) is a Japanese two-row malting barley cultivar (*H. vulgare* ssp. *vulgare*). A total of 93 near isogenic lines (NILs; BC_3_F_2_) were also developed from crosses between H602 (donor parent) and KNG (recurrent parent). To break the strong dormancy of H602 or its derivatives for planting, the grains were treated with 1% (v/v) hydrogen peroxide overnight at room temperature before sowing. Barley plants were grown in an experimental field in Tsukuba, Japan.

### Germination Tests

Germination percentages were estimated using grains manually threshed from spikes harvested at physiological maturity, dried at 30°C for 7 days, then stored at -30°C. Grains from a harvested spike were sown onto two sheets of No. 2 filter paper (ADVANTEC) in 9-cm Petri dishes containing 4.5 mL distilled water with fungicide (0.0125% (w/v) iminoctadine-triacetate). The dishes were incubated in the dark for 7 days at 15°C in a chamber with 100% relative humidity, after which the germinated and ungerminated seeds were counted and germination percentages were calculated. All germination tests were performed on at least three independent biological replicates (approximately 20–30 grains per spike for each biological replicate).

### Genomic DNA Extraction, PCR, and Sequencing

Genomic DNA was isolated from shoots or leaves using the DNeasy Plant Mini Kit (Qiagen), following the manufacturer’s protocol. The genomic DNA sequences were amplified by PCR using TaKaRa Ex Taq or PrimeSTAR GXL DNA polymerase (Takara), according to the manufacturer’s protocol. The PCR conditions and primer sequences are described in Supplementary Table S1. The amplified fragments were purified using a QIAquick Gel Extraction Kit (Qiagen), then sequenced using a 3730xl-Avant DNA Analyzer (Applied Biosystems) and BigDye Terminator version 3.1 reagents (Thermo Fisher Scientific). The sequences were analyzed using Sequencher version 5.2.4 (Gene Codes Corporation) and the DNASIS Pro sequence analysis software (version 2.1; Hitachi Solutions). The genomic sequence of *Qsd1* from KNG was determined as described in Sato et al. (2016).

### Genotyping and Linkage Map Construction

Frozen extracted genomic DNA samples were sent to the Southern California Genotyping Consortium, Illumina BeadLab, at the University of California, Los Angeles, where they were subjected to an oligonucleotide pooled assay (OPA)-single nucleotide polymorphism (SNP) assay using the 1,536-plex barley OPA1 (BOPA1) detection platform developed by Dr. Tim Close, University of California (Close et al., 2009; Sato and Takeda, 2009). OPA genotyping was performed on the 94 RILs and 93 NILs using the Illumina GoldenGate BeadArray. The seven expressed sequence tag (EST) markers used in this study were selected from a set of 384 core markers from the high-density EST marker map (Sato and Takeda, 2009). Genomic sequence information for the known causal genes (Nakamura et al., 2016; Sato et al., 2016) was used for the marker construction of the two major seed dormancy QTL, *Qsd1* and *Qsd2*. These markers were also used to genotype the RILs and NILs. Detailed information about the EST, *Qsd1*, and *Qsd2* markers is provided in Supplementary Table S1. Genetic maps were constructed using JoinMap 4.1 software (Kyazma^1^; Van Ooijen, 2006) with Kosambi mapping function (Kosambi, 1943).

### QTL Analysis

Germination percentages were measured in 2010, 2011, and 2013 for the 94 RILs derived from a cross between H602 and KNG. QTL analysis was carried out using the simple interval mapping (SIM) method with the linkage map and MapQTL 6 software (Van Ooijen, 2009). To detect QTL, the significant logarithm of odds (LOD) threshold of 2.3 was determined using a permutation test (1,000 repetitions, *p* < 0.05). QTL analysis was also performed with mixed model-based composite interval mapping (MCIM) method using QTLNetwork ver. 2.1 (Yang et al., 2008) to identify the main QTL, and to evaluate epistatic interactions and QTL-by-environment interactions across all tested environments. Threshold *F*-values for an experiment-wise significance level of 0.05 were determined by performing 1000 permutations. Tests to detect QTL were conducted at 1-cM intervals with a window size of 10 cM. A Monte Carlo Markov Chain approach was used to estimate the main and epistatic QTL effects.

### Association Analysis between Phenotypes and Genotypes in the F_2_ Progenies

To evaluate the effect of the chromosome segments corresponding to the dormancy QTL regions, F_1_ plants were made from crosses between three RILs with different H602 chromosome 5H segments in the KNG background. The segregation of chromosome segments was determined for approximately 96 F_2_ plants derived from the F_1_ plants using DNA markers. The germination percentages of the resulting F_3_ grains produced from the F_2_ plants with homozygous H602 or KNG genotypes at these markers were determined. The germination percentages of the H602- and KNG-homozygous F_2_ plants were statistically analyzed using a Student’s *t*-test in Microsoft Excel. If a significant difference was detected, the association between germination percentages and the H602 or KNG genotypes at the DNA markers was assessed to determine whether the segregating chromosome segments of the F_2_ plants contain the grain dormancy QTL.

## Results

### Frequency Distribution of Germination Percentage in RILs

**FIGURE 1:**
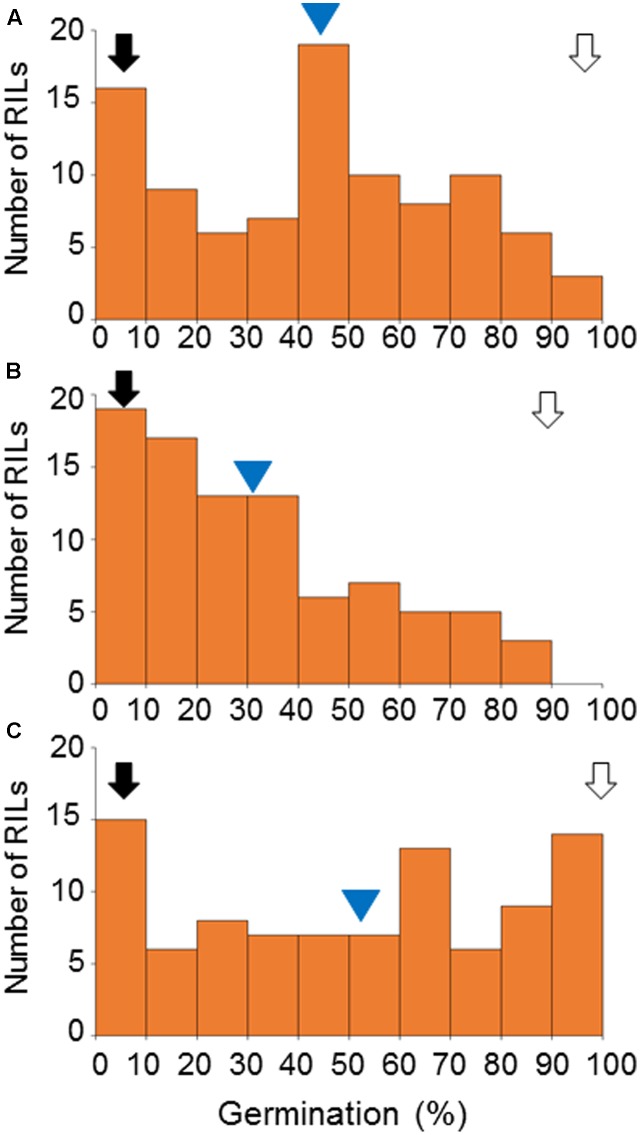
Frequency distribution of the germination percentages of the RILs. Germination percentages for 94, 88, and 92 RILs were measured in 2010 **(A)**, 2011 **(B)**, and 2013 **(C)**, respectively. Black and white arrows indicate germination of H602 and KNG, respectively. Blue triangles indicate mean germination percentage of the RILs.

To examine the distribution and year-to-year variation in germination, we first examined the distribution of germination frequencies in the RIL population grown in three different years. We performed germination tests for the H602 × KNG RILs in 2010, 2011, and 2013 (Supplementary Table S2). The frequency distributions of the RIL germination percentages are shown in **Figure 1**. As expected, the H602 grains showed almost 0% germination in all 3 years while nearly 100% of the KNG grains germinated in 2010 and 2013, which decreased to 90% in 2011. The frequency distributions showed continuous patterns and the overall distributions fluctuated yearly. The mean germination percentages of the RILs were 43, 30, and 51% in 2010, 2011, and 2013, respectively (**Figure 1**). This indicated that the RILs showed the strongest dormancy in 2011, moderate dormancy in 2010, and the lowest dormancy in 2013 (**Figure 1**).

The observed year-to-year variation in the dormancy levels of the RILs might be explained by fluctuations in temperature during grain development, which is thought to be a major factor determining the level of grain dormancy in barley (Benech-Arnold, 2001; Rodríguez et al., 2015). In general, lower temperatures lead to higher levels of dormancy in barley. In our field, the flowering season of the H602 × KNG RILs started around the end of April and the harvest season began at the end of May; therefore, grain developed in the RILs during May. The weather conditions around our fields are monitored and recorded by the Weather Data Acquisition System^2^, and the data from May 2010, 2011, and 2013 are shown in Supplementary Table S3. These data show that the fields had more rainy and cloudy days in May in 2011 compared with 2010 and 2013. In addition, the mean air temperature in May in 2011 was 0.4°C lower than the 17.5°C observed in 2010 and 2013. The mean daily solar radiation was 18.8 MJ/m^2^, 16.2 MJ/m^2^, and 22.1 MJ/m^2^ in 2010, 2011, and 2013, respectively. Therefore, we can estimate that the mean temperature of the spikes during grain development was highest in 2013, intermediate in 2010, and lowest in 2011. This corresponds well with the dormancy levels observed in the RILs; therefore, our results agree with the general rule that lower temperatures during grain development lead to higher acquired levels of dormancy in barley.

### Genetic Map Construction and Analysis of Grain Dormancy QTL

To identify QTL affecting dormancy in this population, we first constructed a genetic map using known markers that are polymorphic in the parents, then used this map to find QTL by two methods, SIM and MCIM. Among the 1,536 SNP markers incorporated in the BOPA1 system, 629 (41%) showed polymorphisms between H602 and KNG. Using genotyping data from the SNP markers, the seven EST markers, and the two major grain dormancy QTL markers (*Qsd1* and *Qsd2*), we constructed a genetic linkage map of the H602 × KNG RILs (Supplementary Tables S4, S5).

#### Simple Interval Mapping

Using the genetic map and the 3 years of germination data, we identified grain dormancy QTL by SIM. The LOD curve patterns for 3 years looked very similar (Supplementary Figure S1). Two QTL were detected above the determined LOD threshold of 2.3, and were designated *Qsd2-OK* and *Qsdw-5H*. An additional LOD peak was observed at the locus of the *Qsd1* marker, and although it was not above the threshold, we designated it *Qsd1-OK*. The calculated parameters of each QTL are summarized in **Table 1**.

**Table 1:** Grain dormancy QTL identified by SIM.

QTL	Year	Flanking marker	Chr.	Peak position (cM)	LOD	PVE (%)	Additive effect (%)
*Qsd1-OK*	2010	*Qsd1*	5H	56.9	2.3	10.9	-9.0
*Qsdw-5H*	2010	*1202*	5H	123.3	3.8	17.3	-12.0
	2013	*1202*	5H	124.3	4.3	19.9	-15.6
*Qsd2-OK*	2010	*Qsd2*	5H	197.5	6.7	28.6	-14.4
	2011	*1509*	5H	196.6	10.3	42.7	-15.5
	2013	*1509*	5H	196.6	12.6	47.4	-22.3


*LOD, logarithm of odds. PVE, phenotypic variation explained by the QTL.*

*Qsd1-OK* was detected at the same location (57 cM) as the causal gene (*AlaAT*) for *Qsd1*, a major dormancy QTL. *Qsd1-OK* explained 11% of the phenotypic variation, and the H602 allele was associated with higher levels of dormancy. Its LOD score is 2.3, just at the determined threshold, and it was detected only in 2010.

*Qsd2-OK* was detected at the *SD2* locus in all 3 years and is flanked by the SNP markers *1509* and *Qsd2*, which are located at 197 cM on chromosome 5H, within a 0.85-cM interval. *Qsd2-OK* explained about 29%, 43%, and 47% of the phenotypic variation in 2010, 2011, and 2013, respectively, and the H602 allele was associated with higher levels of dormancy.

*Qsdw-5H* was detected in the middle of the long arm of chromosome 5H in 2010 and 2013 and is flanked by the SNP markers *1202* and *1168*, which map at 121 and 127 cM, respectively. *Qsdw-5H* explained about 20% of the phenotypic variation in both years, and the H602 allele was responsible for higher dormancy.

#### Mixed-Model-Based Composite Interval Mapping

**FIGURE 2:**
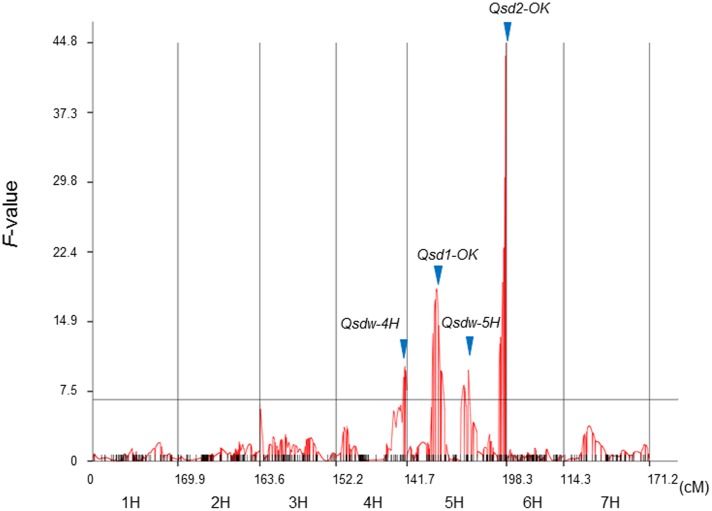
Quantitative trait loci (QTL)-likelihood curves generated by QTLNetwork. Germination data of the RILs with three replications in 3 years, 2010, 2011, and 2013, were used for data processing. Blue arrows indicate the detected QTL locations. The horizontal black line indicates the QTL threshold equivalent to a critical *F*-value of 6.58 (*p* < 0.05). cM: centiMorgan.

In addition to the three QTL detected by SIM, we identified another QTL near the end of the long arm of chromosome 4H by MCIM (**Figure 2** and **Table 2**). This QTL, designated *Qsdw-4H*, is flanked by the SNP markers *79* and *792*, which map at 136 and 138 cM, respectively. *Qsdw-4H* explained about 3% of the phenotypic variation and the H602 allele conferred dormancy at *Qsdw-4H*. The phenotypic variation explained by the QTL and the estimated additive effect values calculated by MCIM (**Table 2**) were a little smaller than the values determined by SIM (**Table 1**). We detected no epistatic interactions between QTL and no additive by environment interactions, indicating that QTL effects were relatively constant through the 3 years.

**Table 2:** Grain dormancy QTL identified by MCIM.

QTL	Chr.	Position (cM)	Flanking markers	Position (cM)	Marker Range (cM)	*F*	PVE (%)	Additive effect (%)	Dormant allele
*Qsdw-4H*	4H	137.7	*79-792*	137.7	135.7–138.0	10.1	3.4	-4.4	H602
*Qsd1-OK*	5H	58.9	*Qsd1-1489*	58.9	56.9–61.9	18.4	6.3	-7.4	H602
*Qsdw-5H*	5H	122.3	*1202-1168*	122.3	121.3–124.3	9.7	13.3	-9.0	H602
*Qsd2-OK*	5H	197.5	*Qsd2-117*	197.5	196.6–197.5	44.8	37.9	-16.7	H602


*QTL, QTL name; Chr., chromosome; Flanking markers, QTL flanking markers; *F*, maximum QTL *F*-value; PVE, phenotypic variation explained by the QTL.*

### Association Analysis of Phenotypes and Genotypes in the F_2_ Progenies

**FIGURE 3:**
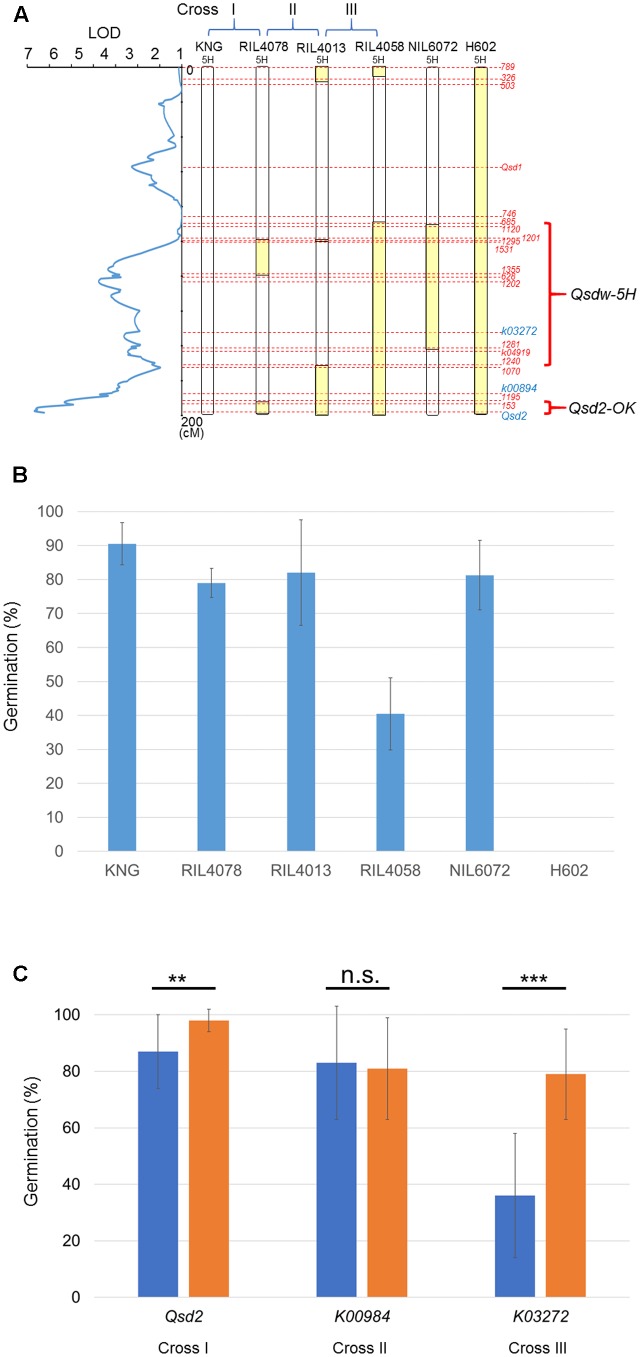
Association analysis between phenotypes and genotypes in the F_2_ progenies. **(A)** Genotypes of the parental lines. Graphical genotypes of chromosome 5H. The results of QTL analysis in 2010 using SIM are displayed on the left. LOD, logarithm of odds. cM, centiMorgan. Yellow bars: H602-homozygous genomic region. Open bars: KNG-homozygous genomic region. Blue letters: DNA markers names used for association analysis. Red letters: DNA marker names. The QTL regions estimated from the association analysis are shown on the right. **(B)** Phenotypes of the parental lines. Germination percentages. The germination test was performed using samples harvested in 2015. Blue bars: mean germination percentages. Vertical bars: standard deviation. *n* = 3 spikes. **(C)** Analysis of the mean germination percentages of F_3_ grains derived from F_2_ plants with homozygous H602 or KNG genotypes at the DNA markers. The germination test was performed using samples harvested in 2015. The F_2_ plants were derived from each cross, Cross I: RIL4078 × KNG, Cross II: RIL4013 × RIL4078, Cross III: RIL4058 × RIL4013. Blue and orange bars show mean germination percentages for F_2_ plants with homozygous H602 or KNG genotypes at the DNA markers, respectively. Vertical bars: standard deviation. *n* = 3 spikes. Italic letters: DNA marker names. Data were analyzed using a Student’s *t*-test. Two asterisks: significant difference at *p* < 0.01. Three asterisks: significant difference at *p* < 0.001. n.s., no significant difference.

To confirm the genomic regions responsible for the QTL, we carried out association analysis using F_2_ plants segregating H602 chromosome segments at the QTL region. The causal gene for *Qsd1* was already identified (Sato et al., 2016) and the effect of *Qsdw-4H* appears to be too small for us to conduct association analysis. For the remaining two QTL, *Qsdw-5H* and *Qsd2-OK*, we used F_2_ plants derived from three crosses: cross I between KNG and RIL4078, cross II between RIL4078 and RIL4013, and cross III between RIL4013 and RIL4058 (**Figure 3A**). To estimate the phenotypic effect of *Qsdw-5H* alone, we also carried out a germination test for NIL6072. RIL4078, RIL4013, RIL4058, and NIL6072 have approximately 7-, 29-, 106-, and 68-cM chromosome segments from H602 on the long arm of chromosome 5H, respectively (**Figure 3A**). H602 and KNG showed about 0 and 90% germination, respectively; RIL4078, RIL4013, and NIL6072 showed similar germination percentages of around 80%; RIL4058 had a germination rate of around 40% (**Figure 3B**).

We compared the mean germination percentages of F_2_ plants with H602- and KNG-homozygous genotypes, determined by the markers *Qsd2*, *K00894*, and *K03272*, which correspond to the segregating chromosome segments in crosses I, II, and III, respectively. We detected a significant difference (*p* < 0.003) in the mean germination percentages of the cross I F_2_ plants with H602- and KNG-homozygous genotypes at the *Qsd2* marker (**Figure 3C**, Supplementary Table S6). This indicates that the 7-cM genomic segment from the *153* marker to the end of long arm of chromosome 5H is involved in the regulation of grain dormancy. This genomic region corresponds to *Qsd2-OK*.

We did not detect a significant difference in the mean germination percentages of F_2_ plants derived from cross II (**Figure 3C**, Supplementary Table S6). This indicates that the approximately 22-cM genomic region between markers *153* and *1070* is not relevant to the regulation of grain dormancy. However, we did detect a strong, significant difference (*p* < 0.001) in the mean germination percentages of the cross III F_2_ plants with H602- and KNG-homozygous genotypes at the *K03272* marker (**Figure 3C**, Supplementary Table S6). This indicates that the 77-cM genomic segment between markers *1070* and *685* is involved in the regulation of grain dormancy. This genomic region corresponds to *Qsdw-5H*.

### Germination of F_1_ Grains

**FIGURE 4:**
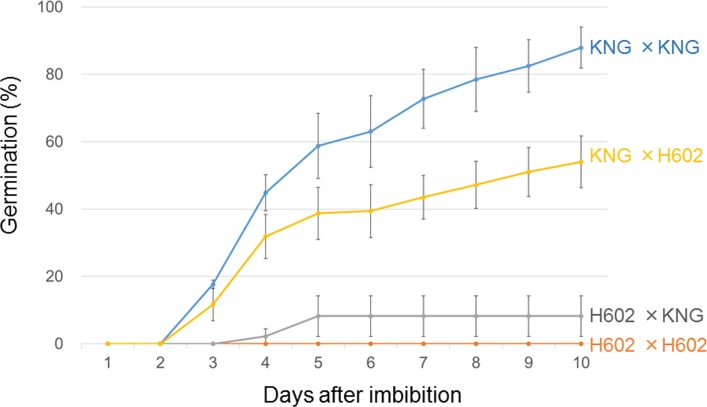
Germination of F_1_ progenies derived from reciprocal crosses between H602 and KNG. Orange line: H602 × H602. Gray line: H602 (as female) × KNG (as male). Yellow line: KNG (as female) × H602 (as male). Blue line: KNG × KNG. Circles: mean germination percentages. Vertical bars: standard deviation. *n* = 3 spikes.

To test for maternal or paternal effects on germination, we tested the germination of F_1_ grains derived from reciprocal crosses between H602 and KNG (**Figure 4**). As the parents H602 and KNG always show strong and weak dormancy, respectively, the F_1_ grains derived from crosses of H602 × H602, and KNG × KNG showed 0 and 88% germination, respectively. However, we detected a large difference in the germinations of the reciprocal crosses. The F_1_ grains derived from a cross with H602 as the female parent and KNG as the male showed 8% germination, which is close to the germination of H602 × H602. By contrast, the F_1_ grains derived from crosses with KNG as the female parent and H602 as the male showed 54% germination, which is an intermediate phenotype between the germinations of the H602 × H602 and KNG × KNG parental crosses. These results indicated the involvement of maternal inheritance in the H602 strong dormancy phenotype.

### Sequence Analysis of *Qsd1* and *Qsd2* in H602 and KNG

To examine whether the known dormancy gene *Qsd1* could be affecting the difference in dormancy between H602 and KNG, we determined the 3787-bp genomic DNA sequence of the *Qsd1* gene in KNG from the start codon to the stop codon (accession no. LC314597). The genomic sequence of *Qsd1* in H602 was reported in a previous study (Sato et al., 2016), which proposed that the amino acid substitution L214F in Qsd1 is the primary cause for the difference between the dormant allele and the less-dormant allele (Sato et al., 2016). Comparison of the deduced amino acid sequences of H602 and KNG showed that both cultivars have an L as the 214th amino acid residue of Qsd1, an allele producing strong dormancy (Sato et al., 2016). This could explain the small effect of *Qsd1-OK* in the H602 × KNG cross. The predicted amino acid sequences of Qsd1 in H602 and KNG differed at positions 288, 371, and 433 (Supplementary Table S7), indicating that these amino acids might contribute to the small effect of *Qsd1-OK*. Comparison of the deduced amino acid sequences of KNG and the malting barley cultivar HN showed that only the 214th amino acid residue was different; HN has F and KNG has an L (Supplementary Table S7). This is consistent with previous results that showing the L214F substitution is a major determinant for the effect of *Qsd1*.

The genomic sequences of *Qsd2* (*MKK3* gene) of H602 and KNG were previously reported (Nakamura et al., 2016). The deduced amino acid sequences of H602 and KNG differed at positions 232, 350, and 383 (Supplementary Table S8). These amino acid substitutions might be related to the large effect of *Qsd2-OK*. However, H602 has the same amino acid residues as Az at these three positions and previous research (Nakamura et al., 2016) revealed that these amino acid substitutions are not the primary cause for *Qsd2-AK*; rather, the N260T substitution is the primary cause for *Qsd2-AK*.

## Discussion

### Dormancy QTL between Wild Barley H602 and the KNG Cultivar

Here, we identified *Qsd2-OK* at the *SD2* locus as a major dormancy QTL for wild barley. *Qsd2-OK* and *Qsd2-AK* appear to be located at the same genetic position, suggesting that both QTL could have the same causal gene. The causal gene for *Qsd2-AK* was already identified as *MKK3* (Nakamura et al., 2016). MKK3 transmits signals by phosphorylation in mitogen-activated protein kinase cascades (Ichimura et al., 2002) and participates in abscisic acid signal transduction (Danquah et al., 2015; Matsuoka et al., 2015), which plays important roles in many developmental and physiological processes, including dormancy (Finkelstein et al., 2008; Nonogaki, 2014; Rodríguez et al., 2015). A single-nucleotide substitution in exon 7 of *MKK3*, which replaces an adenine (A) in the non-dormant allele with a cytosine (C) in the dormant allele (A779C), was found to be the causal sequence polymorphism of *Qsd2-AK* (Nakamura et al., 2016). This SNP results in a non-synonymous amino acid substitution at the evolutionarily conserved 260th amino acid [asparagine (N) to threonine (T)], reducing the kinase activity of MKK3. Therefore, the mutated MKK3 likely cannot efficiently transmit signals for germination and the mutation delays germination and confers the dormant phenotype. The *MKK3* allele of H602 does not have this mutation (Supplementary Table S8, Nakamura et al., 2016), demonstrating that *Qsd2-OK* and *Qsd2-AK* have different causal sequence polymorphisms. Similarly, the dormant lines in which *SD2* was previously detected (Steptoe, Triumph, and TR306) do not have the A779C SNP, again suggesting a different causal sequence polymorphism (Nakamura et al., 2016). Comparison of the predicted amino acid sequences of MKK3 found three amino acid residues that differ in H602 and KNG: V232L, G350R, and D383N in H602 and KNG, respectively (Supplementary Table S8, Nakamura et al., 2016). However, none of these amino acids is evolutionally conserved. Dormant and non-dormant cultivars have the V232, G350, and D383 found in H602, indicating no correlation to the strong dormancy phenotype of H602. In the same way, because dormant and non-dormant cultivars have the R350 and N383 alleles also found in KNG, these changes seem not to be related to the non-dormancy phenotype of KNG. The L232 in the kinase domain is specific to KNG; however, previous kinase assays showed that MKK3 with L232V (as well as R350G and N383D) have similar kinase activities to the KNG MKK3, indicating that L232 has no effect on MKK3 kinase activity in KNG (Nakamura et al., 2016). These results suggest the amino acid differences in MKK3 between H602 and KNG cannot explain the effect of *Qsd2-OK*. Therefore, we need to consider other possibilities for the explanation, such as a potential difference in *MKK3* gene expression or other causal genes that might be located at the vicinity of *MKK3*, such as barley homologs of *PM19-A1* and *A2*, the abscisic acid-induced Wheat Plasma Membrane 19 family genes, which have been proposed as causal genes for *SD2* (Barrero et al., 2015). In this study, we narrowed down the genomic region containing *Qsd2-OK* to a 7-cM sequence. Further narrowing of the genomic region could lead to the identification of the causal gene for *Qsd2-OK*, and will help elucidate the complex regulatory mechanisms involved.

Previously, analyses of dormancy QTL in barley revealed an interesting relationship between *SD1* and *SD2*; either *SD1* or *SD2* is always detected as the major dormancy QTL; in other words, if one is detected, then the other is either not detected or detected as a minor QTL (Gong et al., 2014). This is also true in the case of wild barley H602, although *Qsd1* is thought to be a principal determinant of the differences in dormancy between wild and cultivated barley, and within cultivated barley (Sato et al., 2009). In this study, the effect of *Qsd1-OK* was much smaller than the effect of *Qsd1* previously detected using the HN and H602 DH population. The H602 *Qsd1* allele is common between the two populations and therefore, this difference must be due to a difference in the genetic background between HN and KNG. The genomic sequence of KNG *Qsd1* revealed that KNG Qsd1 and H602 Qsd1 have the same L214 allele, indicating that KNG *Qsd1* might have a similar effect on dormancy to H602 *Qsd1*, but a different effect from HN *Qsd1*. This could explain why the effect of *Qsd1-OK* is smaller than the effect of *Qsd1* from the HN and H602 population. Qsd1 has three more amino acid polymorphisms, in addition to L214F, between H602 and KNG: C288Y, T371I, and M422V (T371I and M422V are polymorphic in KNG and HN; Supplementary Table S7). Sato et al. (2016) mentioned that difference at these three amino acid residues might affect the dormancy level; thus, these differences might result in the small effect observed for *Qsd1-OK*.

We detected another QTL, *Qsdw-5H*, in the middle of the long arm of chromosome 5H. This QTL was not reported in the previous analysis using the HN and H602 population (Hori et al., 2007). Previous studies reported another QTL adjacent to the *SD2* locus (Ullrich et al., 2009; Hickey et al., 2012; Gong et al., 2014); however, *Qsdw-5H* is likely to be located farther from *SD2* than the previously identified QTL. *Qsdw-5H* therefore seems to be a novel dormancy QTL. Our association analysis of Cross III showed that the phenotypes of F_2_ plants that have *Qsdw-5H* and *Qsd2-OK* H602 dormant alleles can clearly be distinguished from the phenotypes of F_2_ plants that have the *Qsdw-5H* KNG non-dormant allele and the *Qsd2-OK* H602 dormant allele (**Figure 3C**, Supplementary Table S6), indicating that we may be able to narrow down the region further to identify the causal sequence polymorphism of *Qsdw-5H*.

Previously, a study using the HN and H602 population detected a dormancy QTL near the end of the long arm of chromosome 4H (Hori et al., 2007). The LOD score was 4.7, the phenotypic variation explained was 5.1%, and its position was between the EST markers *k03067* and *k00136*. We compared the position of the QTL with that of *Qsdw-4H* using the HN/H602 linkage map that was constructed from data of BOPA1 and EST markers (Sato and Takeda, 2009). In the linkage map, *k03067* and *k00136* are located at 112.2 and 129.7 cM, and the flanking markers *79* and *792* for *Qsdw-4H* are at 129.6 and 136.4 cM (Supplementary Table S9). Thus, the *Qsdw-4H* region seems to overlap with the region of the previously identified QTL, suggesting that these two QTL might be the same. This indicates that, although they explained only a small amount of the phenotypic variation, they are real QTL. Therefore, among the four QTL we detected in this study, three of them likely were commonly detected in the HN and H602 population and the H602 and KNG population.

*Qsdw-4H* and *Qsdw-5H* seemed to not be detected within the germplasm from cultivated barley used in the QTL analysis previously reported. Since genetic variation has continued to be reduced by domestication and modern plant breeding (Tanksley and McCouch, 1997), the *Qsdw-4H* and *Qsdw-5H* H602 dormant alleles might have been lost from modern cultivars through bottlenecks imposed by these processes, but could be identified by comparing malting barley with a wild barley accession.

### Maternal Inheritance of the H602 Strong Dormancy Phenotype

The germination test of F_1_ plants derived from the reciprocal crosses showed that the strong dormancy of H602 was maternally inherited. If the cytoplasmic inheritance of organelles were responsible, their DNA should be involved in the inheritance of the strong dormancy trait in H602. The RILs we used all have H602 cytoplasm (Senthil and Komatsuda, 2005), but since not all the RILs displayed the strong dormancy of H602, cytoplasmic inheritance cannot explain the maternal effect of dormancy inheritance. The following three possibilities can explain the maternal inheritance:

(1) The involvement of maternal tissues, such as the testa (seed coat) and pericarp, of grains in the regulation of dormancy. Seed color/pigmentation has been suggested to affect dormancy (Debeaujon et al., 2000; Flintham, 2000). For example, a transcription factor gene, *Hvmyb10* maps to chromosome 3H, regulates proanthocyanidin accumulation in the testa of developing grains, and was reported to affect grain dormancy in barley (Himi et al., 2012).
(2) Genomic imprinting leading to the preferential expression of either maternal or paternal alleles. The expression of maternal-specific alleles leads to maternal inheritance. In plants, expression of imprinted genes occurs primarily in the endosperm (Gehring, 2013; Pires and Grossniklaus, 2014), which is an important tissue for the regulation of germination and dormancy. In cereals, for example, following the release of the hormone gibberellin from germinated embryos, the outermost layer of the endosperm, the aleurone, synthesizes and secretes hydrolytic enzymes that degrade the starch reserves in the endosperm to nourish the growing seedling (Sun and Gubler, 2004). In Arabidopsis, the endosperm directly regulates dormancy; the endosperm of dormant seeds continuously synthesizes and releases abscisic acid toward the embryo to prevent embryonic growth (Lee et al., 2010). Moreover, a recent study reported that several genes related to germination are maternally expressed in Arabidopsis, suggesting that imprinted genes could implement the maternal inheritance of dormancy levels (Piskurewicz et al., 2016).
(3) A gene expression dosage effect resulting from the different numbers of parental genomes in the endosperm tissue, which contains two maternal genomes and one paternal genome.

From the results of the present study, it is not possible to determine which of these possibilities, or which combination of possibilities, causes this maternal inheritance. The future identification of the causal genes for the four QTL might allow us to address this question.

The F_1_ grains from the reciprocal cross KNG × H602 showed 54% germination, which is intermediate between those of the parental crosses KNG × KNG and H602 × H602 (88 and 0%, respectively). Thus, the germination of the F_1_ heterozygote was 10% higher than the average germination (44%) of the two homozygous parental crosses. Finding the explanation for this is also difficult because it does not result from the simple bi-allelic segregation of a gene locus; we need to consider four QTL and the maternal effect involved in regulating the germination of the F_1_ grains. Previous studies showed the dormant alleles of H602 and Az are recessive and the non-dormant alleles of HN and KNG are incompletely dominant in *Qsd1* and *Qsd2-AK* (Sato et al., 2009; Nakamura et al., 2016). Taking these results into account, and if we do not consider the maternal effect for the KNG × H602 cross, the simplest explanation could be that the four QTL might be incompletely dominant with dominance that skews toward non-dormancy or no dominance, and thus the F_1_ grains showed intermediate germination percentages; however, further work is needed to analyze the dominance effect of each QTL.

### Future Perspective

Recently, a high-quality reference genome assembly for barley was released (Mascher et al., 2017), which will accelerate the map-based cloning of QTL genes, including *Qsd2-OK* and *Qsdw-5H*. As demonstrated in this study, we could narrow down the genomic regions for the QTL with an association analysis; further advances may enable us to identify the causal genes for the QTL. The identification of these genes will contribute to basic and applied science, enhancing our understanding of the genetic regulatory mechanisms of seed dormancy, and can be expected to offer opportunities for crop improvement, both in barley and its close relatives, to confer the appropriate level of grain dormancy and avoid pre-harvest sprouting.

## Author Contributions

HM, MS, and KS performed the research and analyzed the data. TK designed the research. MP and SN designed the research, performed research, analyzed the data, and wrote the article.

## Conflict of Interest Statement

The authors declare that the research was conducted in the absence of any commercial or financial relationships that could be construed as a potential conflict of interest.

## Footnotes

1 http://www.kyazma.nl/

2 http://www.naro.affrc.go.jp/org/niaes/aws/weatherdata.html
