# Supplementary material for: Quantitative Trait Loci and Maternal Effects Affecting the Strong Grain Dormancy of Wild Barley (Hordeum vulgare ssp. spontaneum)
Source: Front Plant Sci. 2017 Oct 30;8:1840. doi: 10.3389/fpls.2017.01840 (PMC5674934; doi:10.3389/fpls.2017.01840)
Supplement: Supplementary file 3 [file Table_3.DOCX]

**TABLE S3| Weather conditions in the field in Tsukuba in May**

|  | 2010 | 2011 | 2013 |
| --- | --- | --- | --- |
| Mean temperature (°C) | 17.5 | 17.1 | 17.5 |
| Number of hours of sunshine | 187.4 | 86.7 | 110.3 |
| Mean daily solar radiation (MJ/m^2^) | 18.8 | 16.2 | 22.1 |
| Amount of precipitation (mm) | 104.5 | 215.5 | 48.5 |

The data are available from the Weather Data Acquisition System of Institute for Agro-Environmental Sciences, NARO (http://www.naro.affrc.go.jp/org/niaes/aws/weatherdata.html).
