# Supplementary material for: Quantitative Trait Loci and Maternal Effects Affecting the Strong Grain Dormancy of Wild Barley (Hordeum vulgare ssp. spontaneum)
Source: Front Plant Sci. 2017 Oct 30;8:1840. doi: 10.3389/fpls.2017.01840 (PMC5674934; doi:10.3389/fpls.2017.01840)
Supplement: Supplementary file 6 [file Table_6.DOCX]

**TABLE S6| Mean germination percentages of F_3_ grains derived from F_2_ plants with H602- or KNG-homozygous genotypes at the DNA markers, analyzed using Student’s *t*-test.**

1. Cross I

| Cross | # of  repeats | *p*-value of *t*-test | Genotype of  *Qsd2* | # of  plants | Germination (%) | |
| --- | --- | --- | --- | --- | --- | --- |
|  |  |  |  |  | Mean | STDEV |
| RIL4078×KNG | 1 | 0.00094 | H602 | 20 | 87 | 13 |
|  |  |  | KNG | 29 | 98 | 4 |
|  | 2 | 0.00246 | H602 | 20 | 88 | 12 |
|  |  |  | KNG | 29 | 97 | 4 |
|  | 3 | 0.00026 | H602 | 20 | 82 | 14 |
|  |  |  | KNG | 29 | 96 | 9 |

F_2_ plants with a homozygous genotype at the marker were examined.

Red figures indicate a significant difference at *p*<0.01.

1. Cross II

| Cross | # of  repeats | *p*-value of *t*-test | Genotype of *K00984* | # of  plants | Germination (%) | |
| --- | --- | --- | --- | --- | --- | --- |
|  |  |  |  |  | Mean | STDEV |
| RIL4013×RIL4078 | 1 | 0.81342 | H602 | 15 | 83 | 20 |
|  |  |  | KNG | 28 | 81 | 18 |
|  | 2 | 0.98528 | H602 | 14 | 85 | 17 |
|  |  |  | KNG | 29 | 85 | 14 |
|  | 3 | 0.07816 | H602 | 12 | 75 | 15 |
|  |  |  | KNG | 28 | 63 | 24 |

F_2_ plants with a homozygous genotype at the marker were examined.

(C) Cross III

| Cross | # of  repeat | p-value of t-test | Genotype of *K03272* | # of  plants | Germination (%) | |
| --- | --- | --- | --- | --- | --- | --- |
|  |  |  |  |  | Mean | STDEV |
| RIL4058×RIL4013 | 1 | 0.00000 | H602 | 18 | 36 | 22 |
|  |  |  | KNG | 27 | 79 | 16 |
|  | 2 | 0.00000 | H602 | 18 | 24 | 18 |
|  |  |  | KNG | 27 | 76 | 19 |
|  | 3 | 0.00001 | H602 | 18 | 57 | 20 |
|  |  |  | KNG | 27 | 85 | 12 |

F_2_ plants with a homozygous genotype at the marker were examined.

Red figures indicate a significant difference at p<0.01.
