# Supplementary material for: Quantitative Trait Loci and Maternal Effects Affecting the Strong Grain Dormancy of Wild Barley (Hordeum vulgare ssp. spontaneum)
Source: Front Plant Sci. 2017 Oct 30;8:1840. doi: 10.3389/fpls.2017.01840 (PMC5674934; doi:10.3389/fpls.2017.01840)
Supplement: Supplementary file 7 [file Table_7.DOCX]

|  | Amino acid position | | | | |
| --- | --- | --- | --- | --- | --- |
|  | 214th | 288th | 371th | 422th | 477th |
| H602 | L | C | T | M | T |
| KNG | L | Y | I | V | T |
| HN | F | Y | I | V | T |
| Az | L | Y | I | M | A |
| Amino acid residues in other plant species | L or V | F, P, S or Y | I, T or V | A, K, S, T or V | A, P, D, T, S, or C |

**TABLE S7| Comparison of the different amino acid residues in Qsd1.**

Amino acid residues are presented by one-letter code.

Red letters indicate the causal amino acid substitution for the dormancy QTL *Qsd1* detected using DH lines from HN and H602 (Sato et al. 2016).

The variation of amino acid resides is reported in Sato et al. (2016).

The accession# of the Az Qsd1 gene: LC325498.
