# Supplementary material for: Quantitative Trait Loci and Maternal Effects Affecting the Strong Grain Dormancy of Wild Barley (Hordeum vulgare ssp. spontaneum)
Source: Front Plant Sci. 2017 Oct 30;8:1840. doi: 10.3389/fpls.2017.01840 (PMC5674934; doi:10.3389/fpls.2017.01840)
Supplement: Supplementary file 8 [file Table_8.DOCX]

|  | Amino acid residue position in MKK3 | | | |
| --- | --- | --- | --- | --- |
|  | 232nd | 260th | 350th | 383rd |
| H602 | V | N | G | D |
| KNG | L | N | R | N |
| HN | V | N | R | N |
| Az | V | T | G | D |
| Amino acid residues in other plant species | V or I | N | A, D, E, G, K, P, Q, R, S, or T | N or D |

**TABLE S8| Comparison of the different amino acid residues in MKK3.**

Data for the comparison of the amino acid residues among four cultivars originally presented in Nakamura et al. (2016).

Amino acid residues are presented by one-letter code.

Red letters indicate the identified causal amino acid substitution for the dormancy QTL *Qsd2-AK* detected using RILs from Az and KNG (Nakamura et al. 2016).

The 260^th^ N is the evolutionarily conserved amino acid residue.

Az: Japanese 6-rowed dormant barley cultivar ‘Azumamugi’
