# Supplementary material for: Quantitative Trait Loci and Maternal Effects Affecting the Strong Grain Dormancy of Wild Barley (Hordeum vulgare ssp. spontaneum)
Source: Front Plant Sci. 2017 Oct 30;8:1840. doi: 10.3389/fpls.2017.01840 (PMC5674934; doi:10.3389/fpls.2017.01840)
Supplement: Supplementary file 10 [file Presentation_1.PPTX]

## Slide 1
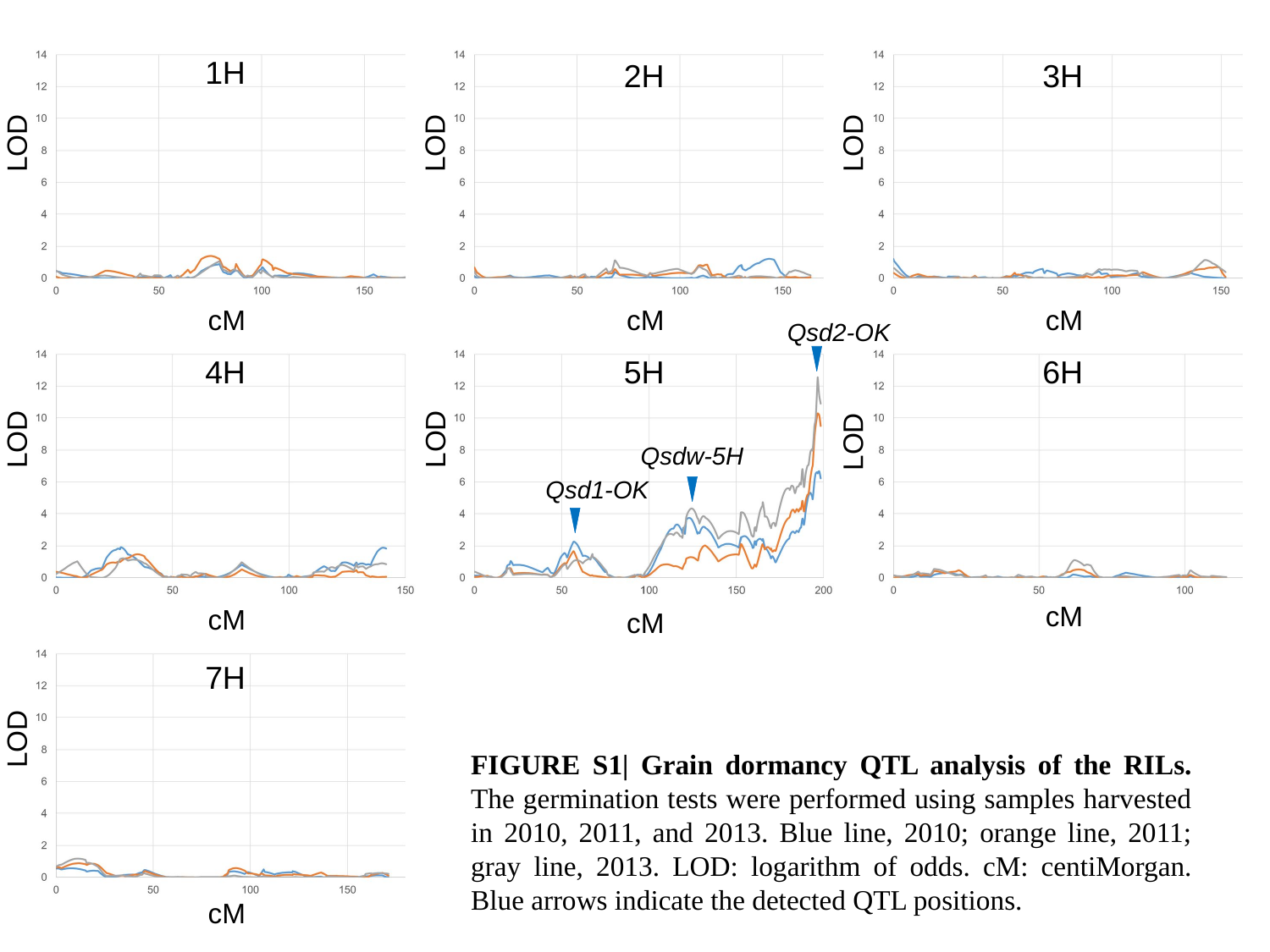

1H
2H
3H
LOD
LOD
LOD
cM
cM
cM
Qsd2-OK
4H
5H
6H
LOD
LOD
LOD
Qsdw-5H
Qsd1-OK
cM
cM
cM
7H
LOD
FIGURE S1| Grain dormancy QTL analysis of the RILs. The germination tests were performed using samples harvested in 2010, 2011, and 2013. Blue line, 2010; orange line, 2011; gray line, 2013. LOD: logarithm of odds. cM: centiMorgan. Blue arrows indicate the detected QTL positions.
cM
